# A comparative analysis depicting the disease characteristics and phylogenetic signature of human cytomegalovirus infection in Human Immunodeficiency Virus 1 seropositive patients with end-organ retinitis and gastro-enteric diseases

**DOI:** 10.1038/s41598-022-11727-2

**Published:** 2022-05-10

**Authors:** Aroni Chatterjee, Debsopan Roy, Sumit Mukherjee, Hiya Ghosh, Agnibha Maiti, Rivu Basu, Nilanjan Chakraborty

**Affiliations:** 1 ICMR-NICED Virus Laboratory, ICMR-National Institute of Cholera and Enteric Diseases, Kolkata, GB4, ID & BG Hospital Campus, Dr. S.C Banerjee road, Beliaghata, Kolkata, West Bengal 700010 India; 2 Azrieli Faculty of Medicine, Bar-Ilan University, Safed, Israel; 3 Department of Endocrinology and Metabolism, IPGMER & SSKM Hospital, Kolkata, 700020 India; 4 Department of Medicine, IPGMER & SSKM Hospital, Kolkata, 700020 India; 5 Department of Community Medicine, R.G. Kar Medical College and Hospital, Kolkata, 700004 India

**Keywords:** Immunology, Molecular biology, Diseases, Risk factors

## Abstract

During advanced HIV infection, Human Cytomegalovirus (HCMV) has been proven to produce devitalizing end-organ diseases (EOD). The interactive co-existence of HIV and HCMV has been reported by many researchers and has been suggested to be linked with a more aggressive disease state. This study has been designed to bring forward an assessment of the clinical risk factors capable of defining the conditions of HCMV induced retinitis and gastro-enteric diseases among HIV1 seropositive patients. We also intended to analyse the phylogenetic variation if any, among the infecting virus types inducing the two separate clinical conditions. The patients were arranged in three different groups; (Group 1 with 26 individuals and group 2 and group 3 with 25 individuals each) based on their current status of HIV and HCMV infections. Serum ELISA, qualitative and quantitative detection of HCMV DNA, Real time mRNA expression study, sequencing, and phylogenetic analysis were performed. All statistical analyses and graphs were exercised using relevant software. We found that in HIV patients with HCMV induced end-organ diseases the components of the CXCL9, 10, 11-CXCR3 chemokine pathway is highly expressed with significant differences existing among patients with retinitis and gastrointestinal disease. We found that the gL gene sequences from the retinitis (HR) group clustered almost separately from that of the gastroenteritis (HG) group in the phylogenetic tree. It may be suggested that a form of natural selection pressure is working on the clinical HCMV strains creating a slight divergence in their phylogenetic lineage thereby helping them adapt to the particular tissue microenvironment they are colonizing.

## Introduction

During advanced HIV infection, Human Cytomegalovirus (HCMV) has been proven to produce devitalizing end-organ diseases (EOD) that includes retinitis, gastrointestinal complications, pneumonitis etc.^1,2^. HCMV mediated retinitis is the most common cause of vision loss in patients with acquired immunodeficiency syndrome (AIDS)^3^. The gastrointestinal (GI) tract is the second most common site of HCMV infection^4^. The interactive co-existence of HIV and HCMV has been reported by many researchers and has been suggested to be linked with a more aggressive disease state^5^. HIV infection causes a rapid shut down of the host’s immune system inducing a state of immunosuppression which thereafter facilitates the infection by opportunist herpes viruses like HCMV^6^. HCMV on the other hand promotes HIV pathogenicity, either by post-transcriptional activation of the HIV proviral DNA, or by stimulating production of inflammatory cytokines which activates the HIV virulent genes^7^. Thus, in a simple sense HCMV acts as a positive cofactor for HIV, resulting in the production of a higher number of HIV viral copies and a more rapid progression to Acquired Immuno-Deficiency Syndrome (AIDS)^8^. It has been reported that in patients with untreated HIV infection, the pro-inflammatory cytokines IL6, TNFα and IL1β arbitrates a systemic acute-phase response to the infection which orchestrates as a series of cascading events governed by the blood monocytes and macrophages leading to the attraction of different innate immune cells at the site of the infection and increased expression of C-reactive protein^9,10^. In specific cases anti-inflammatory cytokines like interleukin-10 (IL-10), IL-4, and transforming growth factor β (TGF-β) are also produced during HIV infection^11^. These cytokines specifically inhibits the inflammatory responses bouight about by the action of the pro-inflammatory cytokines^12^. Besides cytokines, certain chemokines and their receptors are also involved in HIV-HCMV pathogenesis^13^. Studies have shown that CD8+ T lymphocytes produce many potent HIV-suppressive chemokines like MIP-1 alpha, MIP-1 beta etc. which are involved in the control of human immunodeficiency virus (HIV) infection in vivo^14^. In contrast, monocyte chemoattractant protein (MCP)-1 has been observed to up-regulate HIV replication in cultures of CD8- depleted peripheral blood mononuclear cells of HIV-infected patients^15^. The CXCL9, -10, -11/CXCR3 chemokine axis has been another major focus of research in this area, since it regulates the differentiation of naive T cells to T helper 1 (Th1) cells and leads to regulation and migration of immune cells to their focal sites^16^. A lot of researchers have put in effort to elucidate the role of this chemokine axis in the context of the HIV infection^17^. For example, a study has shown that IP10 (CXCL10) produced by Th1 cells significantly stimulates HIV-1 replication in naïve patients^18^ whereas another study has shown that high CXCL10 plasma levels during early infection can be considered as predictive marker of rapid progression to AIDS^19^.

A hallmark of HCMV infection is dissemination to a wide range of host tissue compartments and cell types supporting productive viral infection^20^. Studies from different sources have shown that HCMV is capable of exhibiting significant interhost variability as well as *intra*host diversity (genetic diversity within a single individual) during infection^21^. Mixed infections (Infections with HCMV of diverse genetic variations) accounted for roughly one half of the total HCMV infections among congenitally infected infants and people with HIV/AIDS. Due to the high levels of genotypic diversity in the virus infecting different tissue sites, researchers have tried to understand whether this diversity is a result of a short term spatiotemporal evolution linked to the selection pressure associated with the host tissue environment^22^. Tantalizing evidences has indicated that this intrahost genetic diversity of HCMV helps the virus to differentially adapt in a particular setting by modulating the physiological and immunological microenvironment and largely contributes to the etiology, progression and severity of the disease^23^. Most interhost and intrahost genetic diversity data is based on the analysis of HCMV glycoprotein genes as these regions have been found to show significant variability^24^.

This study has been designed to bring forward a comparative assessment of the haematological and immunological parameters associated with the clinical conditions of HCMV induced retinitis and gastroenteric disease among HIV1 seropositive patients for optimal diagnosis and treatment. By analysing the pattern in the expression of these parameters we will be able to formulate a more advanced defining criterion for monitoring cases of HCMV EODs in AIDS patients thereby generating a therapeutic advantage. Furthermore suitable effort has been put forward to understand the role of specific immunological pathways in the same context. Another critical aspect which we intended to decipher was to investigate whether the HCMV strains infecting the two separate tissue compartments (Retina and gastrointestinal tract) were genetically diverse or not. A positive outcome will provide an experimental support to the long standing hypothesis that the virus infecting at different tissue sites undergo differential genetic evolution to adapt to its surroundings. We chose the HCMV glycoprotein gene, gL (UL115) a major protein involved in the attachment and entry of the virus, for the phylogenetic analysis.

## Results

### Distinctive profiling of HIV1 seropositive patients with HCMV associated end organ diseases (Group 1 patients) based on different baseline parameters

#### Patient’s comparative profile based on demographic and haematological parameters

Out of all the parameters tested only mean CD4+ T cell count, mean concentration of neutrophils and mean haematocrit values showed significant variations among both group 2 (N = 25) and group 3 (N = 25) patients with respect to group 1 (N = 26). All these factors, were significantly lower in case of group 1 patients (HIV1 seropositive with HCMV induced end organ diseases), compared to group 2 (HIV1 and HCMV co-infected without end organ diseases) and group 3 (HIV1 infected without HCMV infection) patients. In all the cases the highest corresponding values were observed in group 3 patients, whereas group 2 patients presented a significantly reduced value compared to group 3. The HCMV viral load was significantly higher in case of group 1 patients when compared to the group 2 patients whereas the HIV viral load was almost similar among both the groups but significantly higher compared to the group 3 patients. The detailed comparative analysis has been provided in Table 1.

**Table 1:** A comparative analysis of the demographic and hematological parameters of the patients measured at the time of admission, differentiating Group 1 (N = 26) from Group 2 (N = 25) and Group 3 (N = 25).

Parameters	Groups	Mean ± SD	Significance	95% CI
Age (years)	Group 1	39.92 ± 10.2	Constant	Constant
	Group 2	40.93 ± 8.85	1.00	− 10.75 to 8.75
	Group 3	40.2 ± 11.16	1.00	− 9.95 to 9.55
BMI	Group 1	19.57 ± 1.6	Constant	Constant
	Group 2	20.08 ± 1.52	1.00	− 2.35 to 1.35
	Group 3	21.68 ± 2.64	0.02	− 3.94 to − 0.0265
Hemoglobin (g/dL)	Group 1	7.33 ± 0.96	Constant	Constant
	Group 2	7.71 ± 1.06	1.00	− 1.53 to 0.78
	Group 3	8.84 ± 1.65	0.007	− 2.66 to − 0.35
Total WBC (× 10^9^/L)	Group 1	3.61 ± 0.93	Constant	Constant
	Group 2	3.85 ± 0.49	1.00	− 1.257 to 0.774
	Group 3	4.04 ± 1.56	0.91	− 1.45 to 0.592
CD4+ T cell count (/mm^3^)	Group 1	62.15 ± 20.24	Constant	Constant
	Group 2	170.9 ± 63.14	0.00	− 148.67 to − 68.83
	Group 3	346.2 ± 45.77	0.00	− 323.9 to − 244.13
Neutrophil (×10^9^/L)	Group 1	0.49 ± 0.24	Constant	Constant
	Group 2	0.72 ± 0.21	0.032	− 0.44 to − 0.015
	Group 3	0.94 ± 0.18	0.00	− 0.656 to − 0.231
Monocytes (×10^9^/L)	Group 1	1.68 ± 0.57	Constant	Constant
	Group 2	1.58 ± 0.41	1.00	− 0.392 to 0.585
	Group 3	1.33 ± 0.41	0.246	− 0.14 to 0.837
Platelets (×10^9^/L)	Group 1	76.9 ± 18	Constant	Constant
	Group 2	88.8 ± 5.69	0.132	− 26.25 to 2.407
	Group 3	96.3 ± 10.9	0.005	− 33.71 to − 5.16
Hematocrit (%)	Group 1	18.52 ± 3.57	Constant	Constant
	Group 2	38.22 ± 6.44	0.01	− 24.32 to − 15.06
	Group 3	51.1 ± 4.98	0.00	− 37.2 to − 27.94
Erythrocyte sedimentation rate (mm/h)	Group 1	97 ± 17.32	Constant	Constant
	Group 2	95.14 ± 8.98	1.00	− 11.74 to 15.46
	Group 3	90.53 ± 9.57	0.721	− 7.132 to 20.07
HIV viral load (×10^3^ copies/mL)	Group 1	327.9 ± 155.8	Constant	Constant
	Group 2	318.44 ± 123.8	1.00	− 114.7 to 133.6
	Group 3	78.9 ± 24.45	0.00	124.7 to 373.1
HCMV viral load (×10^3^ copies/mL)	Group 1	116.84 ± 51.62	Constant	Constant
	Group 2	35.18 ± 7.16	0.00	45.56 to 117.7
	Group 3	–	–	–

Mean ± SD values were calculated and one way ANOVA was performed using Bonferroni method (Post-hoc analysis) for comparing the mean values of each group with respect to that of Group 1.

#### Patient’s comparative profile for serum immunological markers

To test the variable expression pattern of some selective pro and anti-inflammatory cytokines as well as some significant chemokines we performed ELISA using the serum samples of patients belonging to the three distinctive groups. Among all the immunological markers tested, only TGFβ, TNFα, IL1β, IFNγ and CXCL10 presented statistically significant variations among both group 2 and group 3 with respect to the group 1. They were thus selected as critical markers for defining HCMV induced end organ diseases in HIV seropositive patients. Mean concentrations of all of these critical markers were found to be significantly elevated in case of group 1 patients compared to the other two groups. In patients with active HCMV infection, i.e. group 1 and group 2, the mean concentrations of CRP, IL6, IL10 and MCP1 were found to be quite similar but significantly varied from group 3 patients. There was no variation in the mean concentration of MIP1α and IL7 among the patient groups. Detailed comparative analyses for each group have been provided in Table 2.

**Table 2:** A comparative analysis of the different immunological parameters of the patients measured at the time of admission, differentiating Group 1 (N = 26) from Group 2 (N = 25) and Group 3 (N = 25).

Parameters	Groups	Mean ± SD	Significance	95% CI
TGFβ (pg/mL)	Group 1	250.32 + 55.9	Constant	Constant
	Group 2	187.98 + 16.15	0.001	21.96 to 102.73
	Group 3	121.83 + 15.79	0.001	88.11 to 168.87
IL10 (pg/mL)	Group 1	27.12 + 4.95	Constant	Constant
	Group 2	29.03 + 5.98	1.00	− 7.59 to 3.77
	Group 3	35.86 + 7.3	0.001	− 14.43 to − 3.05
IL7 (pg/mL)	Group 1	180.12 ± 23.54	Constant	Constant
	Group 2	190.9 ± 10.38	0.62	− 31.73 to 10.17
	Group 3	184.5 ± 25.22	1.00	− 25.36 to 16.54
IFNγ (pg/mL)	Group 1	23.57 ± 6.83	Constant	Constant
	Group 2	17.84 ± 2.25	0.04	0.204 to 11.26
	Group 3	14.5 ± 5.42	0.001	3.54 to 14.59
TNFα (pg/mL)	Group 1	127.25 + 26.13	Constant	Constant
	Group 2	96.21 ± 7.92	0.001	11.602 to 50.48
	Group 3	40.79 ± 11.92	0.002	67.02 to 105.9
IL6 (pg/mL)	Group 1	32.6 ± 8.04	Constant	Constant
	Group 2	37.6 ± 7.17	0.272	− 12.81 to 2.32
	Group 3	30.05 ± 7.84	1.000	− 5.2 to 9.92
IL1β (pg/mL)	Group 1	123.98 + 38.72	Constant	Constant
	Group 2	91.64 + 7.3	0.016	4.86 to 59.87
	Group 3	45.72 + 9.07	0.001	50.75 to 105.7
CRP (mg/L)	Group 1	19.04 + 2.69	Constant	Constant
	Group 2	18.09 + 2.4	1.00	− 1.64 to 3.55
	Group 3	7.96 + 2.25	0.001	8.48 to 13.68
MCP1 (pg/mL)	Group 1	611.33 + 56.1	Constant	Constant
	Group 2	603.28 + 41.23	1.00	− 45.46 to 61.56
	Group 3	472.55 + 64.23	0.001	85.25 to 192.28
MIP1α (pg/mL)	Group 1	39.05 + 9.49	Constant	Constant
	Group 2	37.64 ± 12.73	1.00	− 8.25 to 11.07
	Group 3	37.11 ± 7.37	1.00	− 7.72 to 11.6
CXCL10 (pg/mL)	Group 1	884.3 ± 58.8	Constant	Constant
	Group 2	569.9 ± 55.7	0.001	263.7 to 364.8
	Group 3	403.15 ± 26.6	0.001	430.6 to 531.6

Mean ± SD values were calculated and one way ANOVA was performed using Bonferroni method (Post-hoc analysis) for comparing the mean values of each group with respect to that of Group 1.

### Comparative profiling of group 1 patients with HCMV induced end organ retinitis and end organ gastro-enteric diseases

To test whether these selected significant markers (Four haematological and Five inflammatory) can help us to distinctively differentiate between the group1 patients with either HCMV induced end organ retinitis or end organ gastro-enteric diseases, a univariate binary logistic regression was performed with the selected markers. 26 patients from group 1 with either end organ retinitis (N = 15) or end organ gastro-enteric disease (N = 11) were chosen as the target population for analysis. The analysis revealed that out of all the selected parameters tested; five parameters, HCMV viral load, TGFβ, TNFα, IL1β and CXCL10 could only significantly predict the prevalence of HCMV induced end organ retinitis among group 1 patients. The mean concentrations for HCMV viral load, TNFα and IL1β were significantly reduced in case of patients with end organ retinitis (HR), whereas the mean concentrations of TGFβ and CXCL10 were significantly higher in case of patients with retinitis. A detailed analysis has been provided in Table 3.

**Table 3:** Binary logistic regression analysis of the selected significant heamatological and immunological factors to ascertain their role while significantly comparing between HCMV induced Retinitis (HR; N = 15) and HCMV induced Gastroenteric disease (HG; N = 11) cases among HIV infected Group 1 patients.

Parameters	Mean ± SD	Significance	Beta coefficient	95% CI	
				Lower	Upper
**CD4+ T-cell count**					
HR	52.3 ± 16.4	0.052	1.069	1.00	1.143
HG	72 ± 19.48				
**Neutrophil**					
HR	0.51 ± 0.22	0.746	0.535	0.012	23.406
HG	0.48 ± 0.27				
**Hematocrit**					
HR	17.95 ± 4.17	0.465	1.102	0.85	1.43
HG	19.1 ± 2.95				
**HCMV viral load**					
HR	92.98 ± 44.5	0.035	1.025	0.998	1.053
HG	140.7 ± 48.8				
**TGFβ (Pg/mL)**					
HR	288.65 ± 35.31	0.016	0.957	0.924	0.992
HG	212 ± 45.66				
**TNFα (Pg/mL)**					
HR	102.96 ± 7.63	0.033	1.652	0.886	1.642
HG	151.55 ± 8.47				
**IL1β (Pg/mL)**					
HR	95.43 ± 11.46	0.022	1.207	0.987	1.475
HG	152.53 ± 34.9				
**CXCL10 (Pg/mL)**					
HR	922.53 ± 47.9	0.02	0.963	0.933	0.994
HG	844.02 ± 41.96				
**IFNγ (Pg/mL)**					
HR	25.28 ± 7.41	0.266	0.921	0.798	1.064
HG	21.96 ± 6.09				

### Determination of cut-off values for significant parameters in predicting HCMV induced end organ retinitis

**Figure 1:**
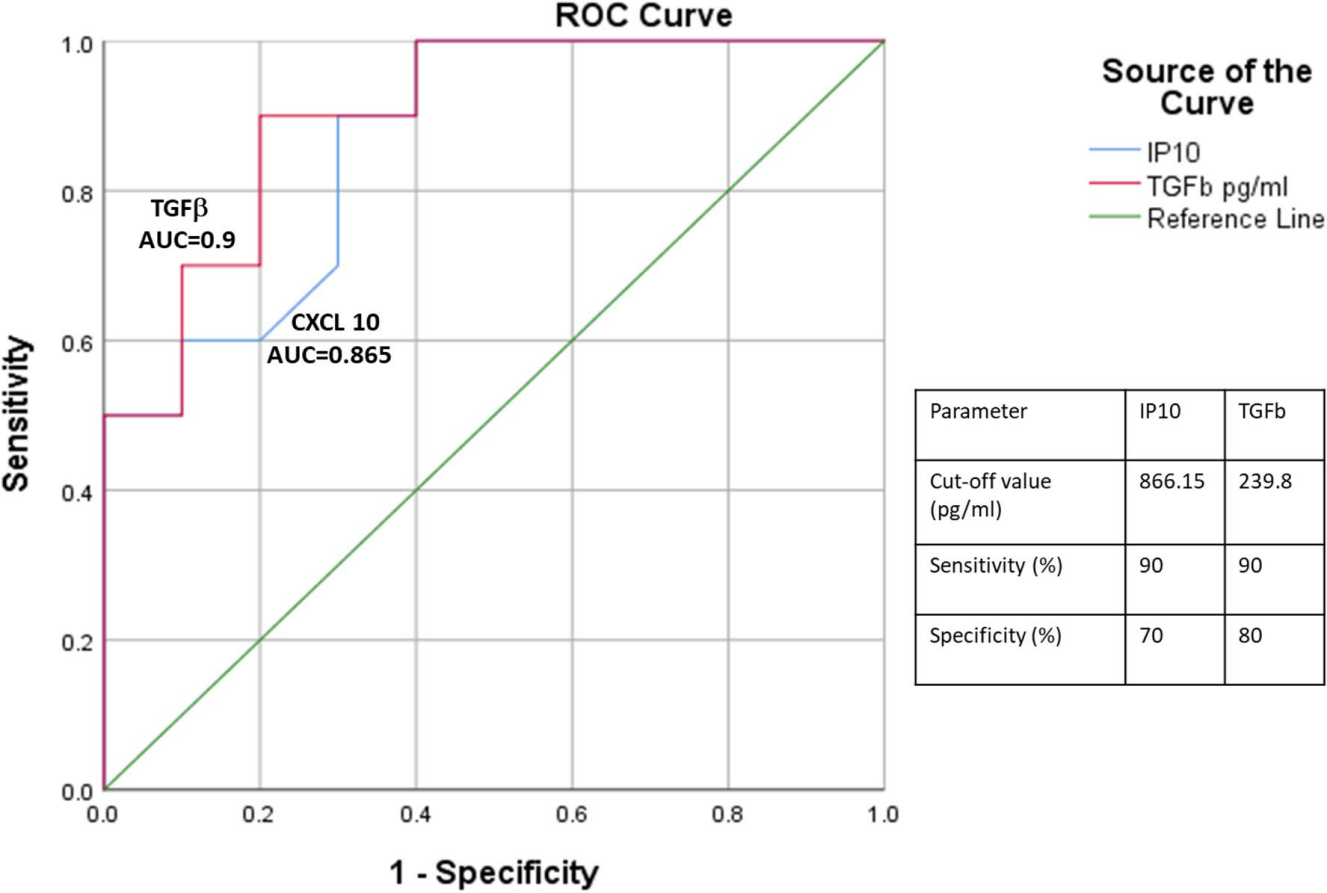
Receiver operating curves (ROC) for identifying the significant biomarkers defining HCMV induced retinitis associated with best predictive accuracy (area under the curve > 0.8).

Receiver-operating characteristic (ROC) curves of the selected models were derived for all the chosen critical biomarkers. The curves were used to evaluate the individual cut-off values prioritizing both highest sensitivity and specificity. Of the 5 predictive biomarkers, namely HCMV viral load, TGFβ, TNFα, IL1β and CXCL10 associated with HCMV induced end organ retinitis or gastro enteric disease in univariate models, only TGFβ and CXCL10 (IP10) demonstrated best accuracy as single biomarkers (AUC 0.900 [95% CI 0.766–1.00] and AUC 0.865 [95% CI 0.707–1.00] respectively). None of the other factors showed good predictive powers for determination. Receiver operating characteristic (ROC) curve for HCMV viral load was found to have a moderate predictive accuracy (AUC 0.750 [95% CI 0.532–0.968]). The ROC curves have been shown in Fig. 1.

### Differential mRNA expression of immunological markers in patients with HCMV induced end organ diseases compared to the other groups

**Figure 2:**
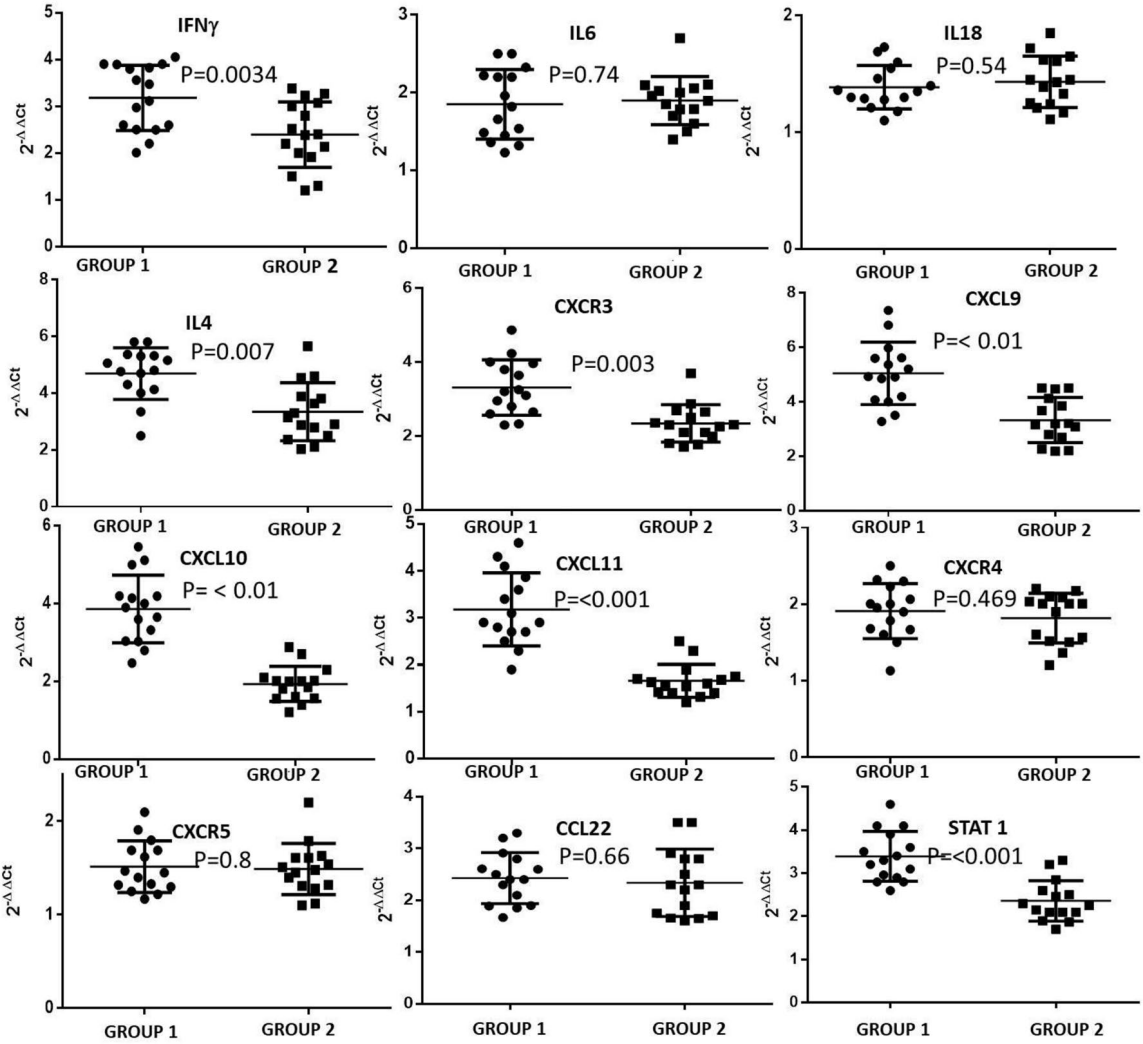
Differential mRNA expression of some selected chemokines and cytokines from patients belonging to the two different groups (N = 15). Their relative mRNA expression ratio with respect to group 3 control group (Baseline expression) in terms of fold change (2^−ΔΔCt^) were estimated by quantitative real time PCR. GAPDH mRNA served as internal control (+ 1 value in Y axis was considered to be the baseline of control, with values greater than 1 suggesting positive fold change and values below 1 upto 0 as negative fold change.

As implicated by the ELISA results the quantitative real time study also showed that the relative mRNA expression ratio for IFNγ was significantly elevated in case of the group 1 (HIV1 seropositive with HCMV induced end organ retinitis or gastroenteric diseases) patients than that of the group 2 (HIV1 and HCMV co-infected without end organ diseases) patients. Group 3 (HIV1 infected without HCMV infection) was chosen as the control group and relative expression of group 1 and group 2 was calculated with respect to this group. Among the members of the CXCL family, the relative mRNA expression of CXCL9, CXCL10, CXCL11 and CXCR3 was found to be significantly higher among the group 1 patients than that of the group 2 patients with respect to the control group. There was almost negligible or very little change in the expression pattern of these markers in group 2 patients when compared to the control group (Group3). The expression pattern for CXCR4 and CXCR5 showed no change among the groups 1 and 2 and also in comparison to the control. Thus the results indicated towards a possible activation of the CXCR3–CXCL9, 10, 11 axes in the group 1 patients. Our hypothesis was strengthened by the fact that both IL4 and STAT1 (Both downstream effectors of the CXCR3-CXCL axis) were also significantly overexpressed in case of the group 1 patients. The macrophage derived chemokine CCL22 showed no significant difference in its expression pattern between group 1 and group 2 patients but its expression was elevated in both cases when compared to the control group. The mRNA relative expression ratio for IL6 and IL18 showed almost no variation among the two groups relative to the control group. Figure 2 provides a clear representation of our findings and the detailed statistical analysis has been provided in the Supplementary Table 2.

### Comparative differential activation of CXCL9, 10, 11-CXCR3 axis in group 1 patients with either HCMV associated end organ retinitis or gastro-enteric diseases

**Figure 3:**
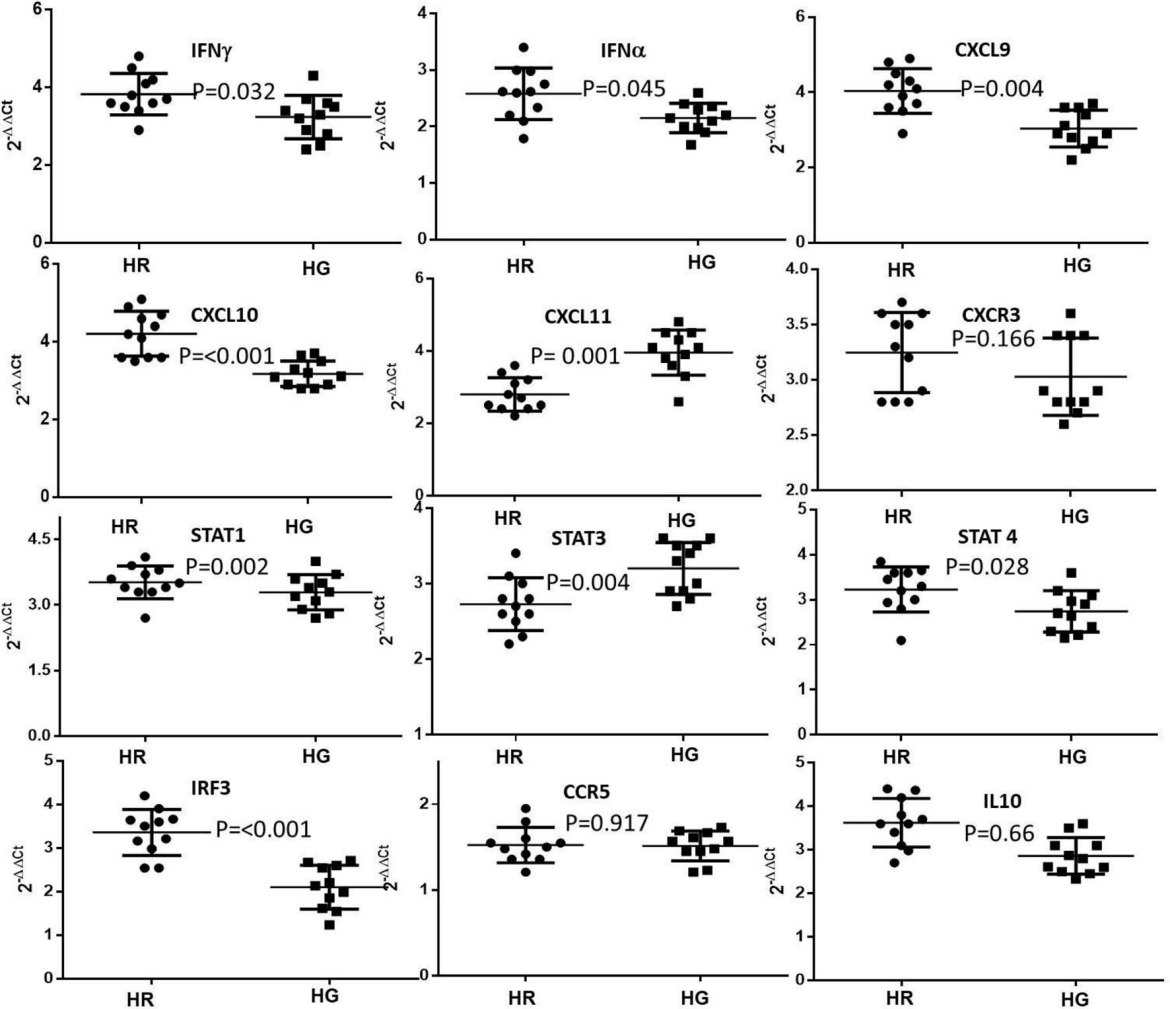
Differential mRNA expression of CXCR3-CXCL axis components from patients belonging to group 1 with either HCMV induced end organ retinitis (HR) or gastro-enteritis (HG) (N = 11). Their relative mRNA expression ratio with respect to group 3 control group (Baseline expression) in terms of fold change (2^−ΔΔCt^) were estimated by quantitative real time PCR. GAPDH mRNA served as internal control (+ 1 was considered to be the baseline of control, with values greater than 1 suggesting positive fold change and values below 1 upto 0 as negative fold change).

To validate whether the CXCR3-CXCL 9, 10, 11 axis is over-expressed similarly in case of retinitis (HR) and gastro-enteric (HG) patients belonging to group 1, we compared the relative mRNA expression of some of the components of the CXCL9, 10, 11-CXCR3 axis along with some downstream effectors. The relative expression of CXCR3 was almost similar among both HR and HG groups, compared to control. The expression of CXCL9 and CXCL 10 was significantly higher in case of HR patients than HG patients, whereas that of CXCL11 was significantly higher in case of the HG patients. There was no significant difference in the expression pattern of IL10 and CC chemokine receptor 5 (CCR5) among both the groups compared to the control group (Group 3). STAT1 and STAT4 were significantly overexpressed among the HR group in comparison to the HG group whereas the expression of STAT3 was significantly higher in case of the HG group. Thus from our observations it can be predicted that in case of the HR group, CXCL9 and CXCL10 are more predominantly expressed, and they furthermore exhibit their downstream action via the STAT1 and STAT4 molecules. In case of the HG group CXCL 11 is the predominant ligand that binds with CXCR3 and exhibits its response via STAT3. The relative expression of IFNγ and IFNα (Both inducers of CXCL 9 and CXCL10) were elevated in case of patients with retinitis. The relative expression of the Interferon Regulatory Factor 3 (IRF3) was also found to be significantly elevated in case of the patients in the HR group. Figure 3 provides a clear representation of our findings and the detailed statistical analysis has been provided in the Supplementary Table 3.

### Phylogenetic analysis of HCMV gL gene sequences from clinically isolated samples

**Figure 4:**
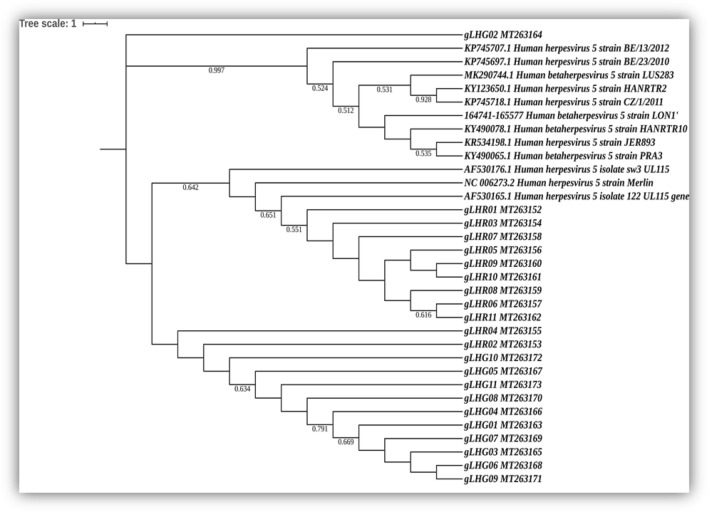
Phylogenetic analysis of the partially sequenced HCMV gL genes from 22 clinical samples belonging to group 1 (11 HR and 11 HG samples) along with 12 standard reference gL gene sequences selected from NCBI database.

The phylogenetic analysis utilizing maximum likelihood method of the partial gL gene sequences from twenty two (22) clinically obtained Group 1 patients {11 with retinitis (gLHR) and 11 with gastrointestinal diseases (gLHG)} and twelve reference NCBI HCMV strains generated a unique tree with most of the gLHR and gLHG gene sequences from the clinical samples being clustered separately. Nine out of the twelve standard gL gene sequences clustered separately with only one clinically isolated sequence, gLHG02-MT263164 remaining in the cluster. The remaining three reference strains, AF530176.1, NC006273.2 and AF530165.1 were found to cluster together with nine clinically isolated gLHR sequences, having a nearest common ancestor in the phylogenetic tree. The remaining two gLHR sequences, gLHR04MT-263155 and gLHR02MT-263153 clustered together with the remaining ten gLHG sequences. The detailed phylogenetic analysis has been provided in Fig. 4.

### Sequence and structure-based analysis to explain the distinct phylogenetic pattern of clinical gL gene sequences

To support our findings relevant to the distinctive phylogenetic patterns observed in case of the clinical *gL* gene sequences belonging to the HR (Retinitis) and HG (Gastro-enteric Disease) groups within group1, we performed some analyses to ascertain their effective number of codons (ENc), codon adaptation index (CAI) and mRNA structure conservation. The ENc values of clinical *gL* genes for HR (mean 42.956 ± 4.51) and HG (mean 53.615 ± 2.47) groups were much different and both were significantly higher than the ENc value of the gL genes from the reference HCMV strains (mean 37.835 ± 4.25). Our results indicated that there exists a significant difference in the codon usage bias among the two groups and the ENc value for the HR group was more closely related to that of the reference strains. ANOVA test showed that there was a significant difference between the HR and HG sub-groups (P ≤ 0.001). The influence of natural selection on the *gL* genes was inferred through CAI analysis, which also demonstrated the adaptation of viral genes to their hosts. The mean ± SD values for CAI of clinically isolated *gL* genes were 0.673 ± 0.071 and 0.769 ± 0.054 respectively for the HR and HG groups respectively. The mean ± SD value for CAI of the reference gL gene was 0.638 ± 0.082. The mean values of the CAI differed significantly among the two groups with that of the HR group more closely related to the CAI of the reference group suggesting a closer phylogenetic relationship. The sequences of viral genes with higher CAIs are considered to be evolutionary more preferable over those with lower CAIs and hence more adaptable to their hosts.

Viral mRNA structure in protein-coding genes plays a significant role in the regulatory process and could determine the efficient translation of viral proteins within the host system^25^. Therefore, to understand if there exists any structural variation in the gL mRNA among the HR and HG clinical groups, we have analyzed the conserved local structures between the two groups. Interestingly, we observed that the average mRNA folding energy of gL sequence for reference strains is − 198.81 kcal/mol, while the HR group is − 198.36 kcal/mol, HG group is − 195.58 kcal/mol. Although the mRNA folding energy of the HR group is similar to the reference strains, the folding energy of the HG group is significantly less than the HR group. It has been reported that weaker folding in viral mRNA structure could promote the faster translation of viral protein in the host system^25,26^. Therefore, this could indicate that the weak folding energy of the gL gene in the HG group could favor distinct patterns of protein synthesis in the gastroenteritis (HG) group.

**Figure 5:**
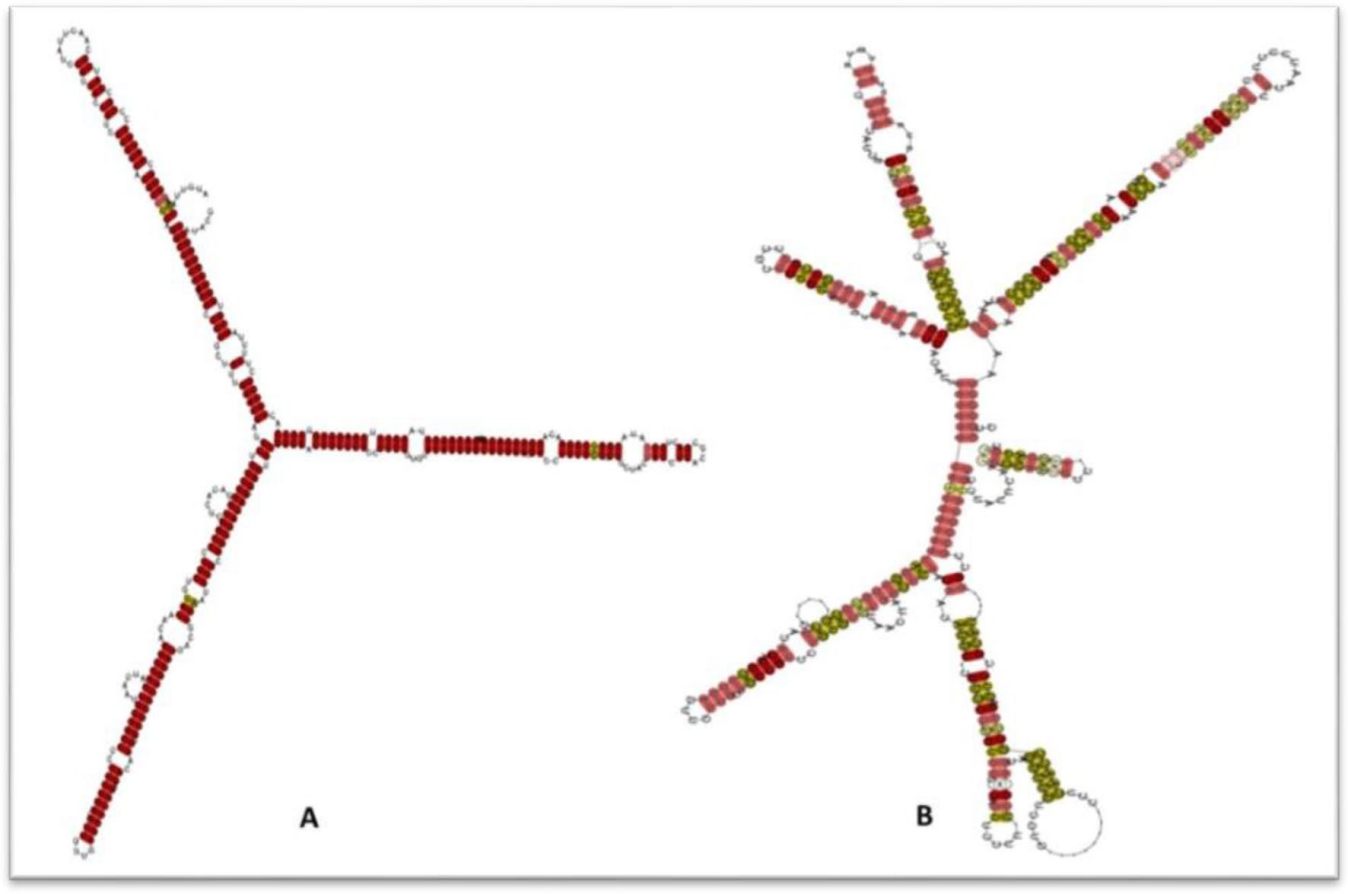
(**A**) Structural classification of the conserved consensus gL mRNA local structures belonging to the clinical group with HCMV induced end organ retinitis (HR). (**B**) Structural classification of the conserved consensus gL mRNA local structure belonging to the clinical group with HCMV induced end organ gastroenteritis (HG).

To understand if there exists any structural variation in the gL mRNA among the HR and HG clinical groups we have analyzed the conserved local structures between the two groups with respect to *gL*. We were able to decipher some significant structural variations in the *gL* mRNAs between the two groups, HG and HR. The consensus *gL* mRNA structures for two groups has been depicted in Fig. 5A,B respectively while their corresponding structural alignments has been provided in Supplementary Figs. 1 and 2, respectively.

## Discussion

In this study we have analysed a large number of haematological and immunological markers, and were able to specify only two immunological markers TGFβ and CXCL10 with exact cut off scores capable of distinguishing between HIV seropositive patient groups with HCMV induced retinitis and gastroenteric diseases. We have identified that in HIV patients with HCMV induced end organ diseases the components of the CXCL 9, 10, 11-CXCR3 chemokine pathway is highly expressed with significant differences among patients with retinitis and gastroenteric disease. When mRNA expression pattern was compared between the two groups it showed that the expression of CXCL9 and CXCL10 was significantly higher in case of retinitis patients whereas that of CXCL11 was higher in case of the patients with gastro-enteric disease. All of these three chemokines utilizes CXCR3 as their receptor and hence the expression of CXCR3 was elevated in both the groups. Our study therefore suggests that in HIV patients with a low CD4+ T cell count, a HCMV co-infection generates an aggravated IFNγ response which thereby leads to the activation of the CXCL9/10/11-CXCR3 chemokine axis. Activation of this chemokine pathway further promotes an acute immune response and inflammation via the Stat proteins; Stat1 and Stat3 in case of the patients with retinitis and Stat4 in case of the patients with gastroenteritis which aggravates the severity of the disease.

Next we wanted to find out whether there exists any phylogenetic variation among the clinical strains causing either retinitis (HR) or gastroenteritis (HG) in the HIV1 seropositive patients. HCMV has already been reported to show major phylogenetic variations among strains infecting different tissue types and for adapting to different physiological and immunological micro-environments^27^. We chose the gene for HCMV glycoprotein L (gL) for this phylogenetic comparison as it is a major envelope glycoprotein that participates in the interaction of the virus with the cell surface markers and also promotes virus-cell fusion^28^. The glycoprotein complexes gH/gL/gO and gH/gL/UL128/UL130/UL131A (Pentamer) are key targets of the human humoral response against HCMV and are required for HCMV entry into different cell types^28,29^. Spontaneous mutations in any of these gene loci might be sufficient enough to generate structural variations in the protein complexes and modify cell tropism of the virus^29^. The specific conserved region of the gL gene that has been amplified and sequenced corresponds to a topological domain on the virion surface involved in virus-cell interaction. Hence any genetic variability the gene shows in this region may point towards a functional variability for the gL protein itself. The phylogenetic tree revealed that the gL gene sequences from the retinitis (HR) group mostly clustered separately from that of the group with gastro-enteric disease (HG). Thus it may be suggested that a form of natural selection pressure is working on the clinical strains creating a divergence in their phylogenetic lineage thereby helping them adapt to the particular tissue microenvironment they are colonizing. To support our findings, we performed ENc and CAI analysis with the clinical and reference gL gene sequences. We found a significant difference in the mean ENc values of the HR and HG strains indicating a differential tissue specific adaptive fitness. The higher mean ENc values of the clinical strains indicate lower codon usage bias in the viral genome which probably helps the viruses to survive within the host by neutralizing the immunogenic pressure of the host. Significant differences in the mean CAI values between the two groups, HR and HG was also observed. A greater mean CAI value probably indicates a higher translational efficiency of the clinical strains resulting in host specific categorical evolution to survive in different physiological and immunological micro-environments. Significant structural variations in the mRNA secondary structures corresponding to the gL gene sequences of clinical HR and HG HCMV strains were also observed. These site-specific variations in the mRNA secondary structures might cause structural or functional alterations to the interacting domains of the viral surface protein thereby differentially modulating their existing affinity for the same cell surface markers and elicit differential immune responses at different tissue sites.

## Conclusion

Our study was able to neatly define two essential markers for identifying clinically active cases of HCMV induced end organ retinitis and gastro-enteric diseases in HIV seropositive patients. The point at which we analysed the different markers was when HCMV induced EODs have already progressed. Hence the critical biomarkers that we have chosen are predictive of the current HCMV EOD state and can significantly differentiate between these two group of patients (HCMV induced Retinitis and HCMV induced Gastroenteritis) at a progressive state of the disease. Furthermore this study also showed the existence of genetic diversity among the HCMV strains causing either end organ retinitis or gastroenteritis among the HIV infected patients which might be the outcome of a possible selection pressure differentially acting on the viral genome helping them to adapt and infect different tissue sites, via distinctive modulation of the micro-environment.

## Materials and methods

### Patient selection

In this study we have chosen HAART naïve HIV1 seropositive patients (CD4+ T cell count < 400 counts/mm^3^) between 20 and 60 years of age with or without HCMV co-infection who visited the ART centre of designated metropolitan hospitals in the span of 2 years. The main focus was on identifying HCMV co-infected (PCR positive) HIV1 seropositive patients presenting either HCMV induced end organ retinitis or gastro-enteric disease at the time of admission. This forms our main target population with a total of 26 individuals collected over a span of one year (15 with HCMV induced retinitis and 11 with HCMV induced gastro-enteric disease). Two other groups containing 25 individuals in each were chosen as controls. The groups were divided in the following manner; Group 1 (HIV1-HCMV co-infected patients who developed either end organ retinitis or gastrointestinal disease), Group 2 (HIV1-HCMV co-infected patients without any symptomatic end organ diseases), Group 3 (HIV1 infected patients without active HCMV infection).

### Inclusion criteria

All HIV1 seropositive patients were chosen by the medical practitioners after carefully monitoring their health conditions, medical reports and clinical profile. To define “proven HCMV end organ disease” like retinitis and gastro-enteric disease, the presence of appropriate clinical symptoms and/or signs were confirmed together with documentation of HCMV in tissue from the relevant organ by histopathology, virus isolation, immunohistochemistry, or DNA PCR. HCMV positive patients were identified by amplifying HCMV DNA in blood using PCR. Molecular diagnoses of other opportunist viruses like HSV, HBV, HCV, HAV, and EBV as well as bacterial, fungal and protozoan cultures etc. were performed and only patients with negative results for all were selected in this study to avoid clinical contradiction.

### Exclusion criteria

Patients who died during the follow-up tenure or for whom complete follow-up data set was unavailable were also later excluded. Moribund patients were excluded from the study.

### Sample collection and patient data collection

5–10 mL of EDTA (Ethylenediaminetetraacetic acid) anti coagulated peripheral blood was collected from patients in vacutainer tubes, processed immediately and serum was separated by centrifugation (1000×*g* for 10 min).

All patients were chosen and data from individual patients were collected by a dedicated medical practitioner directly related to this study. The case notes, charts, investigation results and treatment records of these patients were retrospectively reviewed and statistically analysed by a verified statistician.

### DNA isolation from blood

QIamp DNA blood Mini Kit (Qiagen Inc., Hilden, Germany) was used as per manufacturer’s protocol to isolate whole DNA from the collected blood serum. DNA concentration was measured by measuring OD values using spectrophotometer.

### Qualitative PCR for detection of HCMV

Primers were designed to amplify the UL 83 and gB regions of HCMV genome using primer 3 online software and utilizing standard HCMV strain sequences as reference. The primers were obtained from Eurofins Genomics India Pvt. Ltd. The forward and reverse primers for UL 83 were, 5′-GGG ACA CAA CAC CGT AAA GC-3′ and 5′-GTC AGC GTT CGT GTT TCC CA-3′. The forward and reverse primers for UL 55 (gB) were 5′-GGTCTTCAAGGAACTCAGCAAGA-3′ and 5′-CGGCAATCGGTTTGTTGTAAA-3′. The detailed procedure was performed as per protocol described in one of the previously published study from our laboratory^30^.

### Cytokine ELISA

Serum TGFβ, IL10, TNF*α*, IL-6, CRP, IFNγ, IL1β, IL7, MCP1, MIP1α, and CXCL10 (IP10) levels were measured by using enzyme-linked immunosorbent assay technique kits from G-Biosciences, Geno Technology Inc., USA. All of these assays were designed to detect only human proteins even at very low concentrations. Manufacturer’s protocol was keenly followed while performing each assay.

### Quantification of HCMV viral load

A quantitation standard curve was achieved by using six tenfold serial dilutions of a standard HCMV DNA with known viral load (copies/ml) purchased from ATCC. A conserved partial region of the HCMV UL 75 (gH) gene was amplified in each case and Ct value was measured in a real time PCR instrument (ABI 7500-Applied Biosystems). The detailed procedure was performed as per protocol described in one of the previously published study from our laboratory^30^.

### RNA isolation and real-time polymerase chain reaction

TRIzol reagent (Invitrogen; Thermo Fisher Scientific, Inc.) was used to extract total RNA from the collected whole blood using standard isolation instructions. The isolated RNA was converted into cDNA following standard procedure using primescript 1st strand cDNA synthesis kit (DSS TAKARA Bio India Ltd.). 2 μl of cDNA was used for amplification in a total volume of 20 μl real time PCR reaction mixture with SYBR green dye (TB Green premix ex taq, DSS TAKARA Bio India Ltd.). The quantity of input cDNA was standardized by detecting GAPDH transcripts serving as internal control in the real time PCR assay. Relative quantification was done by determining the real time expression ratios of specific mRNAs using the 2^−ΔΔCt^ method. Primer-3 software was used to manually design the real time primers in the laboratory using and the primers were obtained from IDT technologies INC, India. The reaction conditions and necessary parameters were standardized in the laboratory and have already been published earlier^30^. All the primers that have been used in this study for real time expression have been listed in Supplementary Table 1.

### Statistical analysis

Unpaired t testing and one way analysis of variance (normal distribution) was used for comparing differences between the groups. All results were expressed as mean ± standard deviation, unless otherwise indicated. When the distributions were not normal non-parametric analyses were performed. Comparison between individual groups was done using Bonferroni method along with ANOVA. Statistical significance among two groups was assessed by binary logistic regression. The level of significance was set at 5% in all cases. All P values were two tailed. The SPSS software (version 16.0; SPSS, Inc., Chicago, IL, USA) was used to perform all the statistical analyses. Graph pad prism (ver.6) software was used to prepare all the graphs.

### Gene sequencing

Sequencing primers were designed to partially amplify a conserved region of the HCMV UL 115 (gL) gene using primer 3 software from 22 clinical samples belonging to group 1 (11 patients with retinitis and 11 patients with gastro-enteric disease).The forward and reverse primers are as follows: 5′-GTGAGGTGTTTCAGGGTGACAAGTATG-3′ and 5′-ATTCCTTCACTGCGTTGTACAGGC-3′ respectively. The primers were obtained from Eurofins Genomics India Pvt. Ltd. Thermal cycler was used to amplify the specified region. These crude PCR products were outsourced to Agrigenome Labs Pvt. Ltd for Sanger sequencing. After obtaining the sequences they were analysed and submitted to NCBI gene data bank through sequin (GenBank accession no.s MT263152 to MT263173).

### Phylogenetic analysis of the HCMV gL gene sequences

22 nucleotide sequences corresponding to a conserved region of the HCMV gL gene were obtained from the clinical HCMV strains of Group 1(11 from patients with retinitis and 11 from patients with gastrointestinal disease. 12 complete gL nucleotide sequences belonging to different geographic locations were retrieved from the NCBI RefSeq database as the reference sequences (Available at https://www.ncbi.nlm.nih.gov/). The nucleotide sequences were aligned using MUSCLE^31^ and the poorly aligned regions with more than 20% gaps were trimmed using trimAl^32^. After that, phylogenetic analysis was performed based on Maximum-likelihood (ML) method. The bootstrap analyses were performed with 1000 replicates to determine the robustness of the individual nodes of the phylogenetic tree. Evolutionary analyses were conducted in MEGA-X^33^.

### Analysis of codon adaptation index (CAI)

Codon adaptation index (CAI) is a quantitative measure that predicts the highest relative adaptation of the viruses to their potential host. CAI is calculated using the CAI programmer available in EMBOSS package (http://www.bioinformatics.nl/cgi-bin/emboss/cai)^34^. The reference dataset for *Homo sapiens* were retrieved from the Codon Usage Database (http://www.kazusa.or.jp/codon/).

### Analysis of effective number of codons (ENc)

The degree to which the codon usage deviates from the random selection is defined by the ENc value. The value also depicts the degree of preference for the non-equilibrium use of synonymous codons in the codon family. The ENc value were calculated using the chips programme available in EMBOSS package (http://www.bioinformatics.nl/cgi-bin/emboss/chips)^35^.

### Analysis of mRNA structure of the HCMV gL genes

Here we analysed the potential conserved local mRNA secondary structure of the partially sequenced region of the gL gene and compared the structural patterns between the two groups of clinical strains. LocARNA was used to generate the local structural alignment and consensus structure of the gL gene sequences from clinical and reference strains^36,37^.

### Ethical considerations

The present study and methodologies were approved by the scientific advisory committees (SAC) and certified by Institutional Ethics Committee (IEC) of ICMR NICED, Kolkata as well as all the associated hospitals in accordance with the 1964 Helsinki declaration.

### Patient’s consent

Written informed consents were taken after explaining all associated positive and negative aspects regarding the study to each participating patient in languages at least one of which they understand clearly. Confidentiality of the provided medical and clinical information was maintained properly as per the standard guidelines.

## Supplementary Information

Supplementary Information.

## References

[1] Bowen EF, Griffiths PD, Davey CC, Emery VC, Johnson MA (1996). Lessons from the natural history of cytomegalovirus. AIDS.

[2] Shepp DH, Moses JE, Kaplan MH (1996). Seroepidemiology of cytomegalovirus in patients with advanced HIV disease: Influence on disease expression and survival. J. Acquir. Immune Defic. Syndr..

[3] Lurain NS, Hanson BA, Hotton AL, Weber KM, Cohen MH, Landay AL (2015). The association of human cytomegalovirus with biomarkers of inflammation and immune activation in HIV-1-infected women. AIDS Res. Hum. Retrovir..

[4] Sissons JG, Willis MR (2015). How understanding immunology contributes to managing CMV disease in immunosuprpessed patients: Now and in future. Med. Microbiol. Immunol..

[5] Podlasin RB (2007). CMV infection in HIV-patients. Przegl. Epidemiol..

[6] Whitcup SM (2000). Cytomegalovirus retinitis in the era of highly antirretroviral therapy. JAMA.

[7] Majumder S, Mandal SK, Bandyopadhyay D, Chowdhury SR, Chakraborty PP, Mitra K (2007). Multiorgan involvement due to cytomegalovirus infection in AIDS. Braz. J. Infect. Dis..

[8] George S, Bergin C, Clarke S, Courtney G, Codd MB (2016). Health-related quality of life and associated factors in people with HIV: An Irish cohort study. Health Qual. Life Outcomes..

[9] Casado JL, Arrizabalaga J, Montes M, Martí-Belda P, Tural C, Pinilla J (1999). Incidence and risk factors for developing cytomegalovirus retinitis in HIV-infected patients receiving protease inhibitor theraphy. Spanish CMV-AIDS Study Group. AIDS.

[10] Ramesh K, Gandhi S, Rao V (2015). Clinical profile of human immunodeficiency virus patients with opportunistic infections: A descriptive case series study. Int. J. Appl. Basic Med. Res..

[11] Loo AV, Sujaya S, Peyman M, Florence S, Subrayan V (2011). Retinal manifestations of patients with immunodeficiency virus, a multiethnics study in Malaysia. Int. J. Ophthalmol..

[12] Jabs DA, Ahuja A, Van Natta ML, Lyon AT, Yeh S, Danis R (2015). Long-term outcomes of cytomegalovirus retinitis in the era of modern antiretroviral therapy: Results from a United States cohort. Ophthalmology.

[13] Jabs DA, Van Natta ML, Holbrook JT, Kempen JH, Meinert CL, Davis MD (2007). Longitudinal study of the ocular complications of AIDS: 1. Ocular diagnoses at enrollment. Ophthalmology.

[14] Teoh SC, Wang PX, Wong EP (2012). The epidemiology and incidence of cytomegalovirus retinitis in the HIV population in Singapore over 6 years. Investig. Ophthalmol. Vis. Sci..

[15] Adland E, Klenerman P, Goulder P, Matthews PC (2015). Ongoing burden of disease from HIV/CMV coinfection in Africa in the antirretroviral theraphy era. Front. Microbiol..

[16] Boulougoura A, Sereti I (2016). HIV infection and immune activation: The role of coinfections. Curr. Opin. HIV AIDS..

[17] Yin X, Wang Z, Wu T (2019). The combination of CXCL9, CXCL10 and CXCL11 levels during primary HIV infection predicts HIV disease progression. J. Transl. Med..

[18] Lei J, Yin X, Shang H, Jiang Y (2019). IP-10 is highly involved in HIV infection. Cytokine.

[19] Mehla R, Guha D, Ayyavoo V (2012). Chemokine deregulation in HIV infection: Role of interferon gamma induced Th1-chemokine signaling. J. Clin. Cell Immunol..

[20] Renzette N, Gibson L, Jensen JD, Kowalik TF (2014). Human cytomegalovirus intrahost evolution—A new avenue for understanding and controlling herpesvirus infections. Curr. Opin. Virol..

[21] Sowmya P, Madhavan HN (2009). Analysis of mixed infections by multiple genotypes of human cytomegalovirus in immunocompromised patients. J. Med. Virol..

[22] Coaquette A, Bourgeois A, Dirand C, Varin A, Chen W (2004). Mixed cytomegalovirus glycoprotein B genotypes in immunocompromised patients. Clin. Infect. Dis..

[23] Drew WL, Sweet ES, Miner RC, Mocarski ES (1984). Multiple infections by cytomegalovirus in patients with acquired immunodeficiency syndrome: Documentation by Southern blot hybridization. J. Infect. Dis..

[24] Meyer-König U, Haberland M, von Laer D, Haller O, Hufert FT (1998). Intragenic variability of human cytomegalovirus glycoprotein B in clinical strains. J. Infect. Dis..

[25] Carmi G, Gorohovski A, Mukherjee S, Frenkel-Morgenstern M (2021). Non-optimal codon usage preferences of coronaviruses determine their promiscuity for infecting multiple hosts. FEBS J..

[26] Kiening M, Ochsenreiter R, Hellinger HJ, Rattei T, Hofacker I, Frishman D (2019). Conserved secondary structures in viral mRNAs. Viruses.

[27] Chatterjee A, Mukherjee S, Basu B, Roy D, Basu R, Ghosh H, Bhattacharya M, Chakraborty N (2020). A cross-sectional study exploring disease characteristics and phylogenetic nature of human cytomegalovirus among infected neonates with congenital nephrotic syndrome. Pediatr. Nephrol..

[28] Ciferri C, Chandramouli S, Donnarumma D, Nikitin PA, Cianfrocco MA, Gerrein R, Feire AL, Barnett SW, Lilja AE, Rappuoli R, Norais N, Settembre EC, Carfi A (2015). Structural and biochemical studies of HCMV gH/gL/gO and Pentamer reveal mutually exclusive cell entry complexes. Proc. Natl. Acad. Sci. U. S. A..

[29] Ryckman BJ, Chase MC, Johnson DC (2008). HCMV gH/gL/UL128-131 interferes with virus entry into epithelial cells: Evidence for cell type-specific receptors. Proc. Natl. Acad. Sci. USA.

[30] Chatterjee A, Mukherjee S, Basu B, Roy D, Basu R, Ghosh H, Mishra L, Bhattacharya M, Chakraborty N (2020). Insight into the distinctive paradigm of Human Cytomegalovirus associated intrahepatic and extrahepatic cholestasis in neonates. Sci. Rep..

[31] Edgar RC (2004). MUSCLE: Multiple sequence alignment with high accuracy and high throughput. Nucleic Acids Res..

[32] Capella-Gutiérrez S, Silla-Martínez JM, Gabaldón T (2009). trimAl: A tool for automated alignment trimming in large-scale phylogenetic analyses. Bioinformatics.

[33] Kumar S, Stecher G, Li M, Knyaz C, Tamura K (2018). MEGA X: Molecular evolutionary genetics analysis across computing platforms. Mol. Biol. Evol..

[34] Fuglsang A (2004). The ‘effective number of codons’ revisited. Biochem. Biophys. Res. Commun..

[35] Hu JS, Wang QQ, Zhang J, Chen HT, Xu ZW, Zhu L, Ding YZ, Ma LN, Xu K, Gu YX, Liu YS (2011). The characteristic of codon usage pattern and its evolution of hepatitis C virus. Infect. Genet. Evol..

[36] Kiening M, Ochsenreiter R, Hellinger H-J, Rattei T, Hofacker I, Frishman D (2019). Conserved secondary structures in viral mRNAs. Viruses.

[37] Will S, Joshi T, Hofacker IL, Stadler PF, Backofen R (2012). LocARNA-P: Accurate boundary prediction and improved detection of structural RNAs. RNA.

