# Supplementary material for: A comparative analysis depicting the disease characteristics and phylogenetic signature of human cytomegalovirus infection in Human Immunodeficiency Virus 1 seropositive patients with end-organ retinitis and gastro-enteric diseases
Source: Sci Rep. 2022 May 10;12:7617. doi: 10.1038/s41598-022-11727-2 (PMC9091246; doi:10.1038/s41598-022-11727-2)
Supplement: Supplementary file 1 — Supplementary Information. [file 41598_2022_11727_MOESM1_ESM.docx]

**Supplementary tables**

**Supplementary table 1. The detailed list of primers used for quantitative real time PCR**

| **Gene** | **Forward** | **Reverse** |
| --- | --- | --- |
| **IFNγ** | TTCCTTGATGGTCTCCACAC | GGTCATTCAGATGTAGCGGA |
| **IL 6** | ACCCCCAGGAGAAGATTCCA | CTGAGATGCCGTCGAGGATG |
| **IL 18** | ATGGCTGCTGAACCAGTAGAAG | CAGCCATACCTCTAGGCTGGC |
| **IL4** | CTTTGCTGCCTCCAAGAACA | AATCGGATCAGCTGCTTGTG |
| **CXCR3** | TGAGTGACCACCAAGTGC | AGAGCAGCATCCACATCC |
| **CXCL9** | TGGGCATCATCTTGCTGGTT | ATTTTCTCGCAGGAAGGGCT |
| **CXCL10** | GACAAAATTGGCTTGCAGGA | CCATTCTGATTTGCTGCCTT |
| **CXCL11** | CTTGCTTGCTTCGATTTGGG | CCTGGGGTAAAAGCAGTGAA |
| **CXCR4** | GTCTCAGTGCCCTTTTGTTC | TACAACTCTCCTCCCCATCT |
| **CXCR5** | AGTACAGGCAGCACAGAGAC | GCCTACTCTTCCCTCCATCC |
| **CCL22** | GTTGTCCTCGTCCTCCTTG | AGGAGTCTGAGGTCCAGTAG |
| **STAT1** | CTGTGAAGTTGAGAGATGTGAA | TTCTGGTGCCAGCATTTTTC |
| **STAT3** | GGGAAGAATCACGCCTTCTA | CTAGATCCTGCACTCTCTTCC |
| **STAT4** | CGAATGGCAGAGCAGACAAC | AGCAAAGAGAAGGAACGGCT |
| **IRF3** | TTCATTGTAGGCTCCTGGG | GCACAACCTTGACCATCAC |
| **CCR5** | CCAGTGAGAAAAGCCCGTAA | CTTTTGAAGGAGGGTGGAGT |
| **IL10** | GGCACCCAGTCTGAGAACAG | ACTCTGCTGAAGGCATCTCG |
| **IFNα/IFNA1** | TTCAAAGACTCTCACCCCTG | GTGTAAAGGTGCACATGACG |

**Supplementary table 2**. A comparative analysis of mean relative expression ratio (2^-ΔΔCt^) of different immunological markers quantified using real time PCR. Mean+SD values were calculated and student’s t test was performed to estimate the significance among group 1 and group 2 patients. Group 3 was taken as the control for baseline settings.

| **Markers** | **Mean+SD** | **P value** | **95% CI** |
| --- | --- | --- | --- |
| IFNγ | Group 1- 3.18+0.17 | 0.0034 | -1.29 to -0.3 |
|  | Group 2- 2.394+0.175 |  |  |
| IL6 | Group 1- 1.85+0.115 | 0.74 | -0.24 to 0.33 |
|  | Group 2- 1.89+0.08 |  |  |
| CXCL10 | Group 1- 3.86+0.02 | < 0.01 | -2.45 to -1.41 |
|  | Group 2- 1.93+0.18 |  |  |
| CXCL9 | Group 1- 5.04+0.29 | < 0.01 | -2.4 to -0.96 |
|  | Group 2- 3.33+0.21 |  |  |
| IL4 | Group 1- 4.68+0.24 | 0.007 | -2.07 to -0.6 |
|  | Group 2- 3.34+0.26 |  |  |
| CXCR3 | Group 1- 3.31+0.19 | 0.003 | -1.4 to -0.49 |
|  | Group 2- 2.34+0.13 |  |  |
| CXCR4 | Group 1- 1.9+0.09 | 0.469 | 0.35 to 0.16 |
|  | Group 2- 1.81+0.08 |  |  |
| CXCL11 | Group 1- 3.17+0.2 | <0.001 | -1.97 to -1.6 |
|  | Group 2- 1.66+0.09 |  |  |
| IL18 | Group 1- 1.38+0.05 | 0.54 | -0.11 to 0.19 |
|  | Group 2- 1.43+0.06 |  |  |
| STAT1 | Group 1- 3.39+0.15 | <0.001 | -1.4 to 0.64 |
|  | Group 2- 2.36+0.13 |  |  |
| CCL22 | Group 1- 2.43+0.13 | 0.66 | -0.55 to 0.34 |
|  | Group 2- 2.34+0.17 |  |  |
| CXCR5 | Group 1- 1.51+0.07 | 0.8 | -0.23 to 0.18 |
|  | Group 2- 1.49+0.07 |  |  |

**Supplementary table 3**. A comparative analysis of mean relative expression ratio (2^-ΔΔCt^) of different immunological markers quantified using real time PCR. Mean+SD values were calculated and student’s t test was performed to estimate the significance among the group1 patients with either retinitis (HR) or gastro enteric disease (HG). Group 3 was taken as the control for baseline settings.

| **Markers** | **Mean+SD** | **P value** | **95% CI** |
| --- | --- | --- | --- |
| IFNγ | HR- 3.72+0.162 | 0.032 | -1.07 to -0.1 |
|  | HG- 2.85+0.168 |  |  |
| CXCL10 | Group 1- 4.2+0.174 | <0.001 | -1.4 to -0.61 |
|  | Group 2- 3.18+0.09 |  |  |
| CXCL9 | Group 1- 4.04+0.18 | 0.004 | -1.48 to -0.51 |
|  | Group 2- 3.04+0.15 |  |  |
| CXCL11 | Group 1- 2.88+0.14 | 0.001 | 0.67 to 1.64 |
|  | Group 2- 3.95+0.18 |  |  |
| CXCR3 | Group 1- 3.24+0.109 | 0.166 | -0.53 to 0.09 |
|  | Group 2- 3.03+0.105 |  |  |
| STAT1 | Group 1- 3.52+0.113 | 0.002 | -0.9 to -0.25 |
|  | Group 2- 2.9+0.13 |  |  |
| STAT3 | Group 1- 2.7+0.105 | 0.004 | 0.16 to 0.78 |
|  | Group 2- 3.2+0.106 |  |  |
| STAT4 | Group 1- 3.22+0.15 | 0.028 | -0.9 to -0.05 |
|  | Group 2- 2.74+0.14 |  |  |
| IFNα | Group 1- 2.62+0.138 | 0.045 | -0.7 to -0.09 |
|  | Group 2- 2.13+0.078 |  |  |
| IRF3 | Group 1- 3.36+0.159 | <0.001 | -1.72 to -0.8 |
|  | Group 2- 2.09+0.152 |  |  |
| IL10 | Group 1- 3.6+0.17 | 0.66 | -0.55 to 0.34 |
|  | Group 2- 2.34+0.17 |  |  |
| CCR5 | Group 1- 1.56+0.04 | 0.917 | -0.17 to 0.1 |
|  | Group 2- 1.49+0.07 |  |  |

**Supplementary figures**


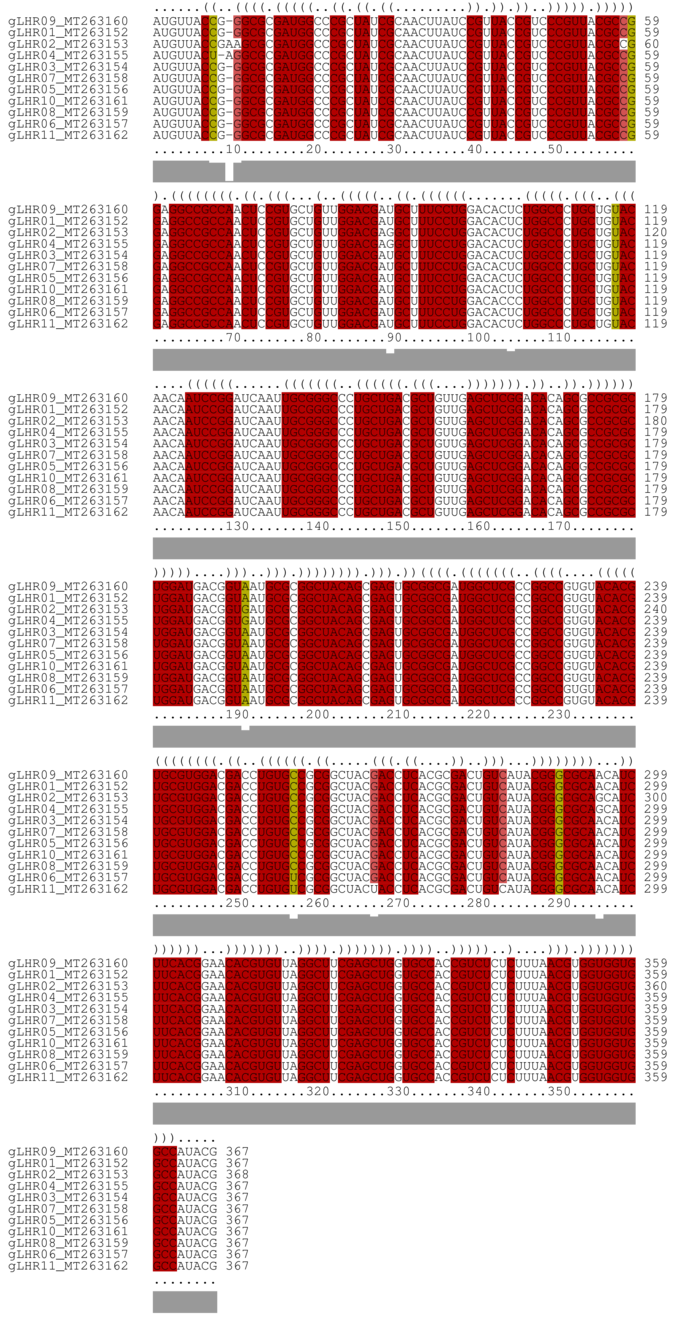


**Supplementary figure 1:** A detailed depiction of the structural alignment for gL gene nucleotide sequences among the HCMV strains belonging to the retinitis group (HR).

**
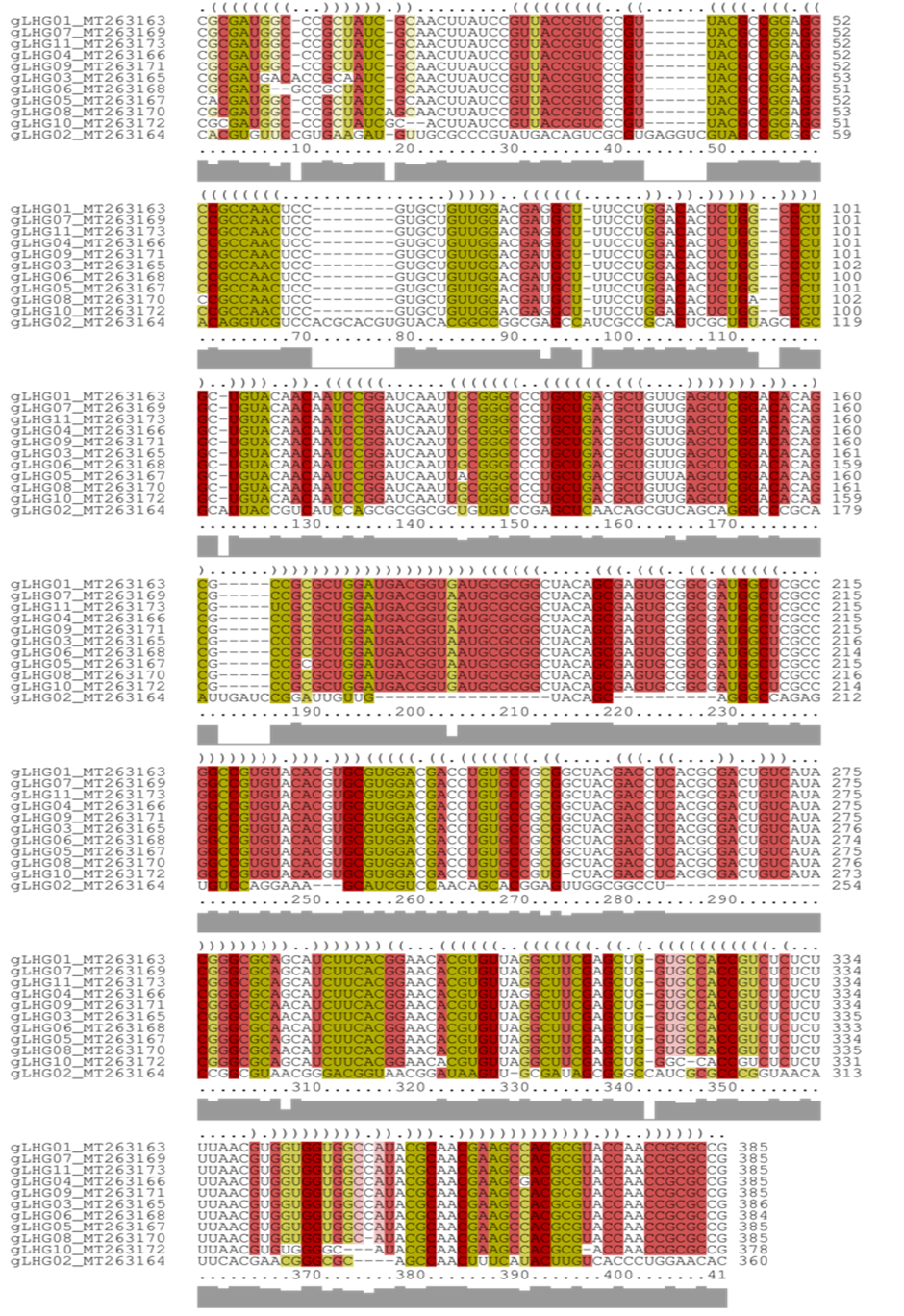
**

**Supplementary figure 2**: A detailed depiction of the structural alignment for gL gene nucleotide sequences among the HCMV strains belonging to the gastroenteritis group (HG).
